# Effects of Meal Fructose/Glucose Composition on Postprandial Glucose Appearance and Hepatic Glycogen Synthesis in Healthy Subjects

**DOI:** 10.3390/jcm10040596

**Published:** 2021-02-05

**Authors:** Cristina Barosa, Rogério T. Ribeiro, Rita Andrade, João F. Raposo, John G. Jones

**Affiliations:** 1 Center for Neuroscience and Cell Biology, University of Coimbra, 3004-504 Coimbra, Portugal; cbarosa@cnc.uc.pt; 2 Education and Research Center, Associação Protetora dos Diabéticos de Portugal (APDP), 1250-189 Lisbon, Portugal; rogerio.ribeiro@apdp.pt (R.T.R.); rita.andrade@apdp.pt (R.A.); joao.raposo@apdp.pt (J.F.R.)

**Keywords:** fructose, deuterated water, glycemic profile, indirect pathway, intestinal gluconeogenesis

## Abstract

Dietary fructose overshadows glucose in promoting metabolic complications. Intestinal fructose metabolism (IFM) protects against these effects in rodents, by favoring gluconeogenesis, but the extent of IFM in humans is not known. We therefore aimed to infer the extent of IFM by comparing the contribution of dietary fructose to systemic glucose and hepatic glycogen appearance postprandially. Twelve fasting healthy subjects ingested two protein meals in random order, one supplemented with 50 g 5/95 fructose/glucose (LF) and the other with 50 g 55/45 fructose/glucose (HF). Sources of postprandial plasma glucose appearance and hepatic glycogen synthesis were determined with deuterated water. Plasma glucose excursions, as well as pre- and post-meal insulin, c-peptide, and triglyceride levels were nearly identical for both meals. The total gluconeogenic contribution to plasma glucose appearance was significantly higher for HF versus LF (65 ± 2% vs. 34 ± 3%, *p* < 0.001). For HF, Krebs cycle anaplerosis accounted for two-thirds of total gluconeogenesis (43 ± 2%) with one-third from Triose-P sources (22 ± 1%). With LF, three-quarters of the total gluconeogenic contribution originated via Krebs cycle anaplerosis (26 ± 2%) with one-quarter from Triose-P sources (9 ± 2%). HF and LF gave similar direct and indirect pathway contributions to hepatic glycogen synthesis. Increasing the fructose/glucose ratio had significant effects on glucose appearance sources but no effects on hepatic glycogen synthesis sources, consistent with extensive IFM. The majority of fructose carbons were converted to glucose via the Krebs cycle.

## 1. Introduction

Over the past few decades, consumption of refined sugar, which includes sucrose and high-fructose corn syrup, has sharply increased worldwide and is implicated in the increased prevalence of obesity and its metabolic complications such as nonalcoholic fatty liver disease (NAFLD) and type 2 diabetes [1,2]. While the glucose and fructose components of refined sugar are isocaloric, the pathophysiology of NAFLD and related complications seems to be accelerated more by fructose than glucose [2]. Thus, there is high interest in understanding how this is linked to the differential metabolism of these sugars by the body.

The liver has long been assumed to be the main site for fructose metabolism [3,4]. However, recent studies in mice have shown a capacity for intestinal fructose metabolism coupled to intestinal gluconeogenesis [4,5]. By this mechanism, fructose is immediately metabolized by intestinal tissues, with glucose and other metabolic products such as lactate and alanine appearing in the portal vein blood [4]. This has two important implications for the metabolism of refined sugar. First, metabolism of fructose by the liver is limited to conditions where the intestinal capacity is exceeded, for example following high sugar intake. Second, to the extent that fructose is converted to glucose by the intestine, intake of a fructose load that is within the intestinal metabolic capacity will result in a portal vein glucose excursion resembling that from a glucose load of the same size. Thus, from the perspective of the portal glucose sensor, β-cell insulin response, and systemic glycemic excursion, fructose and glucose loads of this size are indistinguishable. The activity of intestinal gluconeogenesis appears to be highly species dependent [5], and for humans, the capacity of intestinal fructose metabolism in relation to the amount of refined sugar intake is not well characterized.

Thus, our aim was to apply deuterated water (^2^H_2_O) to determine the sources of systemic glucose appearance and hepatic glycogen synthesis following meal tolerance tests with low and high fructose levels, respectively.

**Figure 1:**
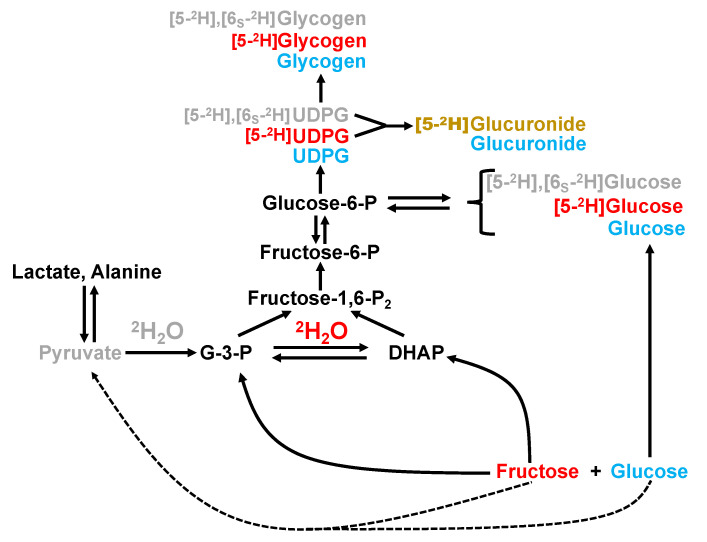
Metabolic scheme relating ^2^H-enrichment in positions 5 and 6 of circulating glucose, UDP-glucose (UDPG), glucuronide and hepatic glycogen with the utilization of fructose and glucose from the test meal. On conversion of fructose to dihydroxyacetone phosphate (DHAP) and glyceraldehyde-3-phosphate (G-3-P), hydrogen exchange mediated by triose phosphate isomerase incorporates ^2^H from ^2^H_2_O into position 5 of glucose and glycogen pathway products, represented in red. In addition, a small amount of fructose may be converted to pyruvate via glycolysis before being incorporated into glucose and glycogen via gluconeogenesis. This possibility also holds for dietary glucose. Passage of these and other gluconeogenic precursors, including lactate and alanine, will generate glucose and glycogen pathway products enriched in both positions 5 and 6*_S_* (represented in grey). Glucose from the meal that is directly absorbed will not be enriched in position 5 or 6 nor will these positions become enriched during subsequent conversion to UDPG, glucuronide and glycogen via the direct pathway (represented in blue). Formation of glucuronide from UDPG results in the loss of the position 6 hydrogens, hence this metabolite informs enrichment of position 5 from all gluconeogenic precursors including fructose (represented in brown). Some metabolic intermediates have been omitted for clarity.

As summarized in Figure 1, the contribution of the meal fructose and glucose components to the appearance of circulating glucose and hepatic glycogen can be estimated by measuring their positional ^2^H-enrichments from ^2^H_2_O [6,7,8]. In humans, the ^2^H-enrichment distribution of the glucosyl components of newly-synthesized liver glycogen can be noninvasively measured via “chemical biopsy” of uridine diphosphate glucose (UDP-glucose) by Paracetamol and analysis of the urinary Paracetamol glucuronide product [9,10]. From this information, the fractional contributions of direct and indirect pathway fluxes to newly-synthesized glycogen can be estimated. Fructose is metabolized to glycogen exclusively via the indirect pathway [8], while dietary glucose is converted to glycogen via both direct and indirect pathways [11,12,13]. Thus, the relative contributions of direct and indirect pathways to hepatic glycogen synthesis are influenced in part by the availability of fructose relative to glucose at the hepatocyte level.

In this study, we compared glycemic excursions and quantified the sources of circulating glucose and hepatic glycogen synthesis in healthy subjects that ingested two meals containing moderate amounts of refined sugar (50 g per meal). In one meal, the sugar had a low fructose/glucose ratio (5/95) while for the other meal, the fructose/glucose ratio was higher, resembling that of high-fructose corn syrup (55/45). Our results reveal a remarkable degree of metabolic flexibility and the possible involvement of intestinal fructose metabolism in the maintenance of postprandial glycemic control when a major proportion of meal glucose was replaced by fructose.

## 2. Experimental Section

### 2.1. Human Studies

**Figure 2:**
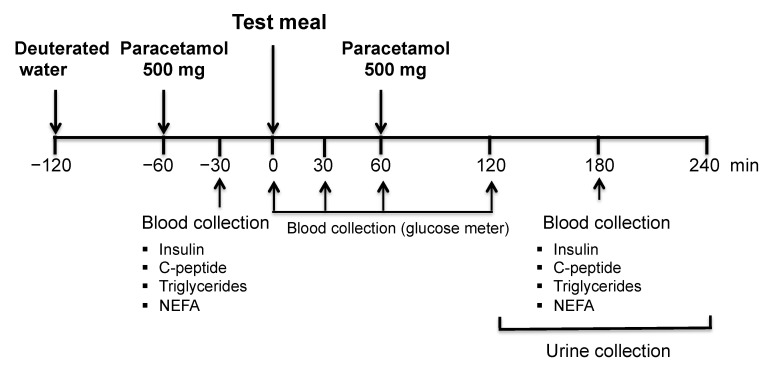
Description of the study protocol.

The study protocol (Project Identification Code 143/2016), summarized in Figure 2, was approved by the Ethics Committee of the Associação Protetora dos Diabéticos de Portugal (Portuguese Diabetes Association) (APDP) and performed after informed consent from the participants.

Twelve healthy subjects (seven males, five females; age: 23–53 year; BMI: 19.8–28.7 Kg/m^2^) underwent the meal studies at two separate occasions. After overnight fasting, the subjects ingested a single loading dose of 99.9% ^2^H_2_O over a 20 min period to attain 0.3% body water (BW) ^2^H enrichment. Drinking water containing 0.3% ^2^H was provided for the study duration in order to maintain body water ^2^H-enrichment. Thirty minutes before the meal ingestion, blood samples were drawn for baseline insulin, C-peptide, triglycerides, non-esterified fatty acids (NEFA). Each subject then ingested a meal composed of 20 g of protein (100% Whey Isolate 5 glutamine, Scitec Nutrition) and 50 g of a fructose/glucose mixture dissolved in 300 mL water. The fructose/glucose proportion of the meals was either 55/45, hereafter referred to as high fructose (HF); or 5/95, hereafter referred to as low fructose (LF). HF and LF meals were ingested in random order with a minimum interval of 6 weeks between each meal to ensure complete washout of ^2^H before the second meal study.

Blood glucose levels were measured immediately before the meal and at 30, 60 and 120 min after the meal. A 30 mL blood sample was collected at 180 min post-meal for assay of insulin, C-peptide, triglycerides and NEFA and also for analysis of blood glucose ^2^H enrichment. For chemical biopsy of hepatic UDP-glucose ^2^H-enrichment, 0.5 g Paracetamol was ingested 1 h before and 1 h after each meal and urine was collected from 2–4 h after the meal.

### 2.2. Blood Biochemical Parameters

Capillary glucose was measured using a standard clinical glucometer (Contour XT, Ascensia Diabetes Care, Lisboa, Portugal). Plasma insulin and C-peptide were determined by the chemiluminescence technique using a Liaison Analyzer (DiaSrin, Stillwater, MN, USA), triglycerides and NEFA were determined by enzimatic assay using an automated clinical biochemistry analyzer (Olympus AU640 Clinical Chemistry System, Beckman Coulter Inc., Brea, CA, USA).

### 2.3. Blood Glucose and Urinary Glucuronide Processing for ^2^H NMR Analysis

Blood glucose was derivatized to monoacetone glucose (MAG), Paracetamol glucuronide was isolated from the urine and derivatized to monoacetone glucuronolactone (MAGLA) and positional ^2^H-enrichments were analyzed by ^2^H NMR as previously described [6]. Body water ^2^H-enrichment was determined by ^2^H NMR from urine analysis as previously described [6,14,15] and it is referred in the text and tables as body water (BW).

### 2.4. NMR Spectroscopy

^2^H NMR spectra were obtained in a 500 MHz Bruker Avance III HD (Bruker BioSpin, Wissembourg, France) equipped with an UltraShieldPlusTM magnet and with a 5 mm ^2^H-selective probe. MAG and MAGLA ^2^H NMR spectra were acquired at 50 °C and a 25 °C, respectively, and using a 90-degree pulse angle, 1.0 sec acquisition time and 0.6 s pulse delay. For determination of BW ^2^H-enrichment, NMR spectra were acquired at 25 °C and using a 22.5-degree pulse angle, 4 sec acquisition time and 8 sec pulse delay. MAG, MAGLA and BW ^2^H-enrichments were calculated from the analysis of the ^2^H spectra using ACD/NMR Processor Academic Edition from ACD/Labs software (Advanced Chemistry Development, Inc., Toronto, On, Canada) www.acdlabs.com, 2015.

### 2.5. Sources of Plasma Gglucose and Hepatic Glycogen Synthesis

Plasma glucose that was derived from the meal absorption or was in circulation before ^2^H_2_O administration (pre-existing) will not be enriched by ^2^H in positions 5 and 6*_S_*. Glucose that was derived from all gluconeogenic substrates, including those feeding directly into the triose-phosphate pools such as fructose and glycerol, is enriched in position 5. Glucose that was derived from gluconeogenic precursors that are metabolized via pyruvate and/or Krebs cycle (for example glutamine or alanine, or pyruvate/lactate derived from fructose) is enriched in both positions 5 and 6*_S_*. On this basis, the sources of systemic plasma glucose following the meals were calculated as follows:

Plasma glucose from gluconeogenesis (%) = (^2^H5_gluc_/^2^H BW) × 100, (1)

Plasma glucose from absorbed or pre-existing glucose (%) = [1 − (^2^H5_gluc_/^2^H BW)] × 100, (2)

where ^2^H5_gluc_ is enrichment of the glucose position 5 hydrogen and ^2^H BW is the enrichment of body water.

The total gluconeogenic contribution was resolved into Krebs cycle and triose-phosphate contributions as follows:

Plasma glucose from Krebs cycle gluconeogenesis (%) = (^2^H6*_S_*_Gluc_/^2^H BW) × 100, (3)

Plasma glucose from Triose-P gluconeogenesis (%) = (^2^H5_Gluc_ − ^2^H6*_S_*_Gluc_)/^2^H BW) × 100, (4)

where ^2^H6_SGluc_ is enrichment of the glucose position 6_S_ hydrogen.

Contributions of direct and indirect pathways to glycogen synthesis via were quantified from the ^2^H-enrichment of the glucuronide position 5. Newly-synthesized UDP-glucose and glycogen formed from glucose via the direct pathway will not be enriched in position 5 whereas glycogen derived via the indirect pathway, which includes contributions from fructose, will be enriched in position 5. It is assumed that the position 5 enrichment of urinary paracetamol glucuronide is equivalent to those of UDP-glucose and newly-synthesized glycogen.

Indirect pathway contribution (%) = (^2^H5_UDPG_/^2^H BW) × 100, (5)

Direct pathway contribution (%) = (1 − (^2^H5_UDPG_/^2^H BW)) × 100, (6)

where ^2^H5_UDPG_ is the ^2^H-enrichment of UDP-glucose position 5 as measured by urinary glucuronide analysis.

### 2.6. Statistical Analysis

All the results are presented as means ± SEM. Parameter comparisons within the same study were made by paired *t*-test while comparisons between studies were made by unpaired *t*-test. Statistical significance was defined as a *p* value of less than 0.05.

## 3. Results

### 3.1. Plasma Biochemical Parameters

**Figure 3:**
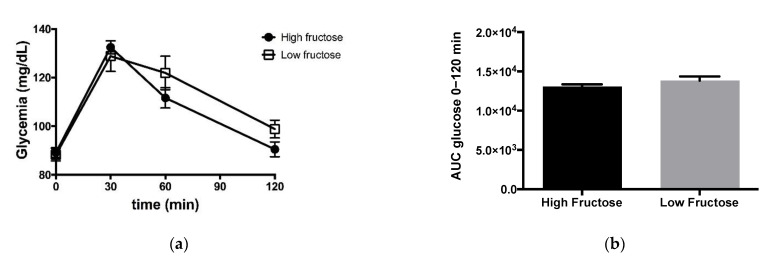
(**a**) Glycemic profile for the high and low fructose studies. Data points are represented as means ± SEM. (**b**) Area under the curve (AUC) for each meal are shown on the right-hand side. AUC values are represented by arbitrary units.

Glycemic profiles after LF and HF ingestion are shown in Figure 3.

Glucose excursions were essentially similar for both meals. Plasma biochemical analyses are shown in Table 1.

Triglyceride, NEFA and c-peptide concentrations measured three hours after the test meal ingestion did not differ from those measured at baseline for both studies. However, for LF, the insulin concentration was significantly lower after the meal compared to baseline. HF showed a similar evolution of insulin levels but the difference between final and baseline levels did not reach statistical significance. While all subjects ingested a standardized meal, we found that there was no significant influence of BMI on any of the biochemical parameters measured either before or after meal ingestion.

### 3.2. Plasma Glucose and Urinary Glucuronide ^2^H Positional Enrichments from ^2^H_2_O

**Figure 4:**
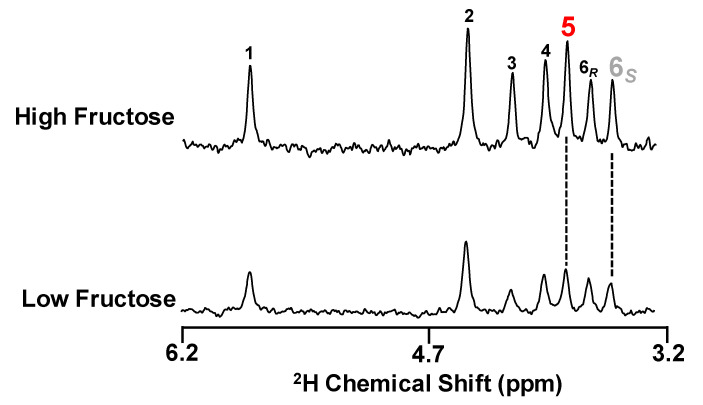
Representative ^2^H NMR spectra of the MAG derivatives of plasma glucose of healthy subjects after ingestion of a meal with 50 g sugar consisting of 55/45 fructose/glucose (High Fructose) or 5/95% fructose/glucose (Low Fructose). The vertical height of each spectrum is scaled to the ^2^H-enrichment of position 5. The numbers above each signal represent the ^2^H position in the plasma glucose, with positions 5 and 6S highlighted in red and gray, respectively.

Representative ^2^H-NMR spectra of MAG derivatives prepared from blood glucose sampled three hours after ingestion of the meals are shown in Figure 4.

The well-resolved ^2^H signals from each of the carbon-bound hydrogens of glucose allowed precise measurement of ^2^H-enrichments in all sites, including those of 5 and 6S which inform gluconeogenic contributions from anaplerotic Krebs cycle substrates and from substrates metabolized directly to triose-P-which can include fructose-to plasma glucose appearance [6] (see Table 2, Equations (1)–(4)). The ^2^H-enrichment of body water was the same for both meals. The ^2^H-enrichments of position 5, as well as that of 6S, were significantly higher for HF compared to LF. Enrichment of the position 1 hydrogen originates from the same gluconeogenic precursors as that of position 6S alongside an additional ^2^H-exchange process mediated by mannose-6-P isomerase that is independent of gluconeogenic activity [16]. Thus, the magnitude of the difference in position 1 enrichment between HF and LF (0.16% and 0.11%) corresponds exactly to that of the 6S position (0.12% and 0.07%). In contrast to plasma glucose, urinary glucuronide enrichment did not show significant differences in ^2^H-enrichment between LF and HF for either position 5 or position 1. Note that glucuronide does not report the 6S hydrogen enrichment of its UDP-glucose precursor since this hydrogen is removed during glucuronide formation. Nevertheless, enrichment of the position 1 hydrogen of glucuronide tracks that of UDP-glucose 6S in an analogous manner to the position 1 and 6S of plasma glucose. Since no differences in glucuronide position 1 enrichment were observed between LF and HF meals, we conclude that the 6S enrichments of UDP-glucose were also not different. This has important implications on the metabolic route taken by the fructose carbons during their conversion to hepatic glycogen.

### 3.3. Sources of Plasma Glucose Appearance and Hepatic Glycogen Synthesis Following LF and HF Meals

**Figure 5:**
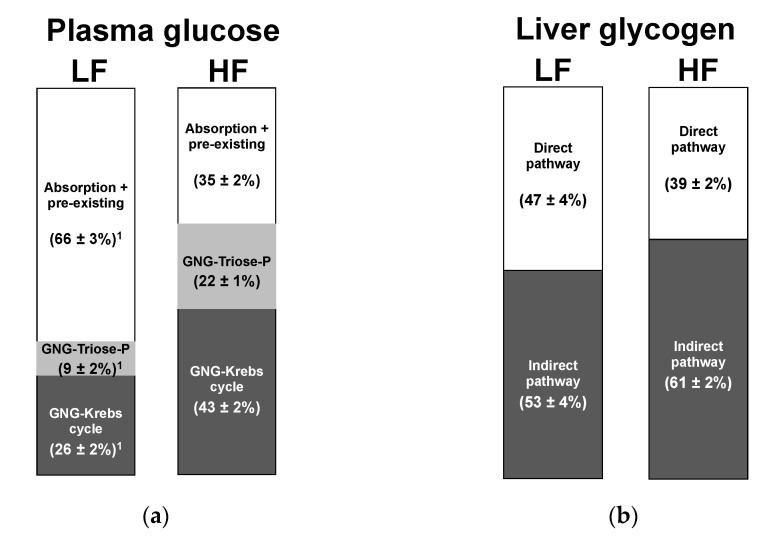
Sources of (**a**) plasma glucose appearance and (**b**) hepatic glycogen synthesis following meals supplemented with 50 g sugar containing 5/95 fructose/glucose (low fructose, LF) and 55/45 fructose/glucose (high fructose, HF). ^1^ *p* < 0.01 versus the corresponding HF values.

Figure 5 shows the sources of plasma glucose appearance and contributions of direct and indirect pathways to hepatic glycogen synthesis (see Table 2, Equations (5) and (6)) as estimated from the ^2^H-enrichment data of positions 5 and 6*_S_* of glucose, and position 5 of urinary glucuronide, respectively.

For the LF meal, two-thirds of plasma glucose was accounted by absorbed glucose and/or any unlabeled circulating glucose that was present before ^2^H_2_O administration. One-third was synthesized via gluconeogenesis, with the largest share of gluconeogenic carbons having passed via the Krebs cycle and a minority derived from substrates directly feeding the triose-P pool, including fructose. For the HF meal, absorbed and/or pre-existing glucose accounted for only one-third of glucose appearance with gluconeogenesis providing the remaining two-thirds. The share of gluconeogenic carbons entering directly the triose-P pool rose significantly, reflecting the conversion of fructose to glucose via the canonical fructokinase → aldolase B → triokinase pathway followed by conversion of the triose-P products to glucose via gluconeogenesis. However, this was accompanied by a substantial increase in gluconeogenesis via the Krebs cycle, which became the largest overall contributor to glucose appearance.

In contrast to the significant differences observed in plasma glucose sources between HF and LF meals, the contributions of direct and indirect pathways to hepatic glycogen synthesis were relatively constant. There was a tendency for a higher direct pathway contribution to glycogen synthesis for LF compared to HF, with a corresponding tendency for a reduced indirect pathway contribution in LF compared to HF. While glucuronide ^2^H-enrichment analysis does not provide resolution of indirect pathway fluxes into Krebs-cycle and Triose-P, as is the case for analysis of the position 5 and 6S of liver glycogen, the fact that the glucuronide position 1 enrichments did not vary between LF and HF suggests that the proportion of carbons feeding the indirect pathway via Triose-P relative to those incorporated via the Krebs cycle was similar for both meals.

## 4. Discussion

Humans have a highly-evolved capacity for dietary glucose and fructose utilization. This includes efficient absorption and metabolism via distinctive transporters and metabolic pathways. While the initial steps of fructose metabolism to triose phosphates are not subject to regulation by insulin, the ultimate fates of the fructose carbons (glucose, glycogen, glycolytic products and lipids) are highly controlled by insulin and other regulators of systemic carbohydrate metabolism such as carbohydrate-responsive element-binding protein ChREBP. In addition, each sugar influences the metabolism of the other. For example, fructose-1-P formed by phosphorylation of fructose stimulates glucokinase activity thereby promoting the phosphorylation of glucose and its conversion to glycogen [17,18,19]. In addition, fructose uptake and metabolism is dependent on the expression and activation of ChREBP, in part mediated by glucose [20,21]. Thus, the physiological effects of fructose ingested alone is markedly different from that ingested alongside glucose. Likewise, the systemic effects of glucose ingestion are highly modified by the presence of fructose. For healthy subjects, ingestion of a pure fructose load resulted in a significantly lower plasma glucose excursion compared to either an equivalent glucose load or a mix of fructose and glucose [22,23,24]. When a 75 g oral glucose load was accompanied by 7.5 g fructose, plasma glucose excursions were reduced compared to that from the glucose load alone with no change in plasma insulin levels [24]. Studies of both isolated rat hepatocytes and livers from intact rats have shown interactions of glucose and fructose on the status of signaling proteins involved in the regulation of hepatic glucose metabolism such as PI3-kinase and GSK3, as well as levels of metabolites that play a primary regulatory role in glycogen synthesis, such as glucose-6-phosphate and ATP [25].

We observed that ingestion of meals containing 50 g of refined sugar with low (5/95) and with high (55/45) fructose/glucose ratios resulted in remarkably similar glycemic excursions. However, the sources of plasma glucose appearance were highly influenced by the meal fructose/glucose levels. With the high fructose/glucose meal, the majority of plasma glucose appearance was sustained by gluconeogenesis, with a significant proportion of the gluconeogenic carbons presumably originating from fructose. Had the fructose been converted to glucose via triose-P, it would have resulted in a large excess of position 5 enrichment relative to that of 6*_S_* (see Figure 1). Such an enrichment pattern was observed for plasma glucose of rats fed a chow diet supplemented with sucrose. In contrast, for the high fructose/glucose meal of our study, the difference in enrichment between the 5 and 6*_S_* positions was far less pronounced. This is consistent with a substantial fraction of fructose carbons being metabolized to glucose via Krebs cycle anaplerosis of pyruvate rather than by the more direct triose-P route. In a study of healthy subjects provided with a fat and protein meal supplemented with ^13^C-enriched fructose alongside an equivalent amount of unlabeled glucose, the appearance of ^13^C-enriched lactate was observed alongside that of ^13^C-enriched glucose [26]. Since this methodology does not distinguish between ^13^C-glucose appearance from ^13^C-fructose via the triose-P or anaplerotic Krebs cycle routes (the Krebs cycle route will result in a portion of the ^13^C-label being lost as ^13^CO_2_ via exchanges at the level of malate, oxaloacetate and fumarate, the proportion of fructose that was converted to glucose via pyruvate anaplerosis relative to that metabolized via triose-P could not be estimated. Our data suggests that at least under the conditions of our study, the dominant route for the conversion of fructose to glucose was via pyruvate anaplerosis.

In the study of Jang et al., where mice were given 0.5 g/kg of a 1:1 glucose/fructose mixture, about 50% of the circulating postprandial glucose was derived from the fructose component of the load [4]. In our HF study, where subjects ingested ~0.6 g/kg of a 55/45 fructose/glucose load, the gluconeogenic contribution to postprandial glucose was 65%, an increase of 30% over that of LF. Assuming that this increase in gluconeogenic contribution was entirely from the increased proportion of fructose in HF vs. LF, we estimate that fructose accounted for about one-third of postprandial glucose appearance and about half of the gluconeogenic contribution to glucose appearance. Some of the variance in these data may reflect the fact that the meal was standardized with each subject receiving different amounts of sugar on a gram/kg basis.

**Figure 6:**
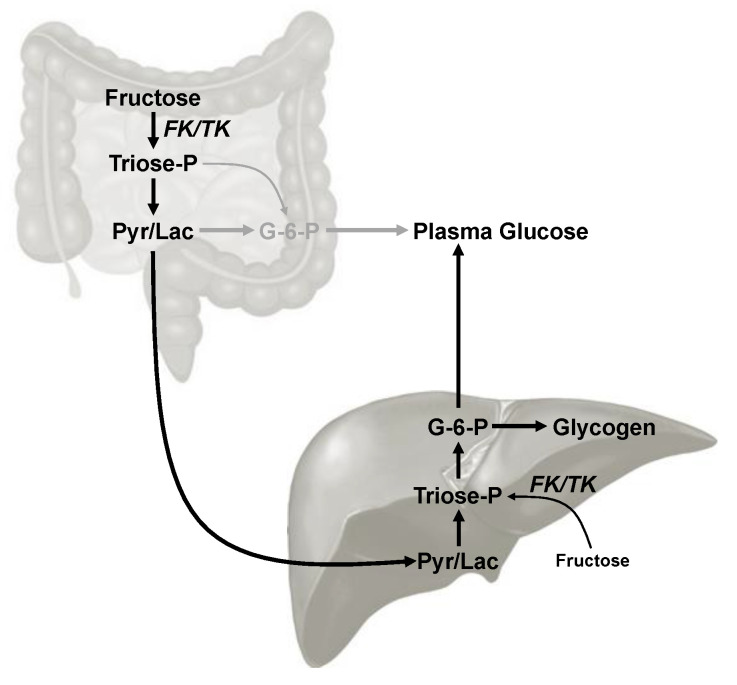
Metabolic model describing intestinal and hepatic routes of fructose catabolism from a protein meal supplemented with 50 g of a 55/45 fructose/glucose mixture corresponding to HFCS-55. Following entry into the glycolytic triose-P intermediates via fructokinase and triokinase (FK/TK), conversion of the pyruvate/lactate intermediate (Pyr/Lac) to glucose can occur by hepatic gluconeogenesis (in black) or by intestinal gluconeogenesis (in gray). Overall, conversion of fructose to glucose-6-P via triose-P is minor in comparison to its conversion via pyruvate/lactate.

As for glucose, the ^2^H-enrichment pattern of hepatic glycogen is also sensitive to the contributions of absorbed glucose, gluconeogenic substrates metabolized via triose-P and gluconeogenic substrates metabolized via anaplerosis [6,8]. For both rats and mice provided with chow supplemented by high levels of sucrose or high-fructose corn syrup in the drinking water, there was a significantly higher contribution of Triose-P sources to hepatic glycogen synthesis compared to rodents fed chow alone as seen by the markedly higher of position 5 enrichment relative to position 6*_S_* [6,7,8]. These observations are consistent with hepatocyte metabolism of fructose to glycogen via Triose-P [7]. In contrast to these animal model observations, the direct and indirect pathway contributions to hepatic glycogen synthesis that we observed in our subjects did not significantly differ between HF and LF. Moreover, there was no indication of a higher triose-P contribution to the indirect pathway of hepatic glycogen synthesis for HF, as suggested by the glucuronide position 1 enrichment data. This suggests that the amounts of fructose metabolized to glycogen by the hepatocytes were similar for both meals, consistent with a substantial proportion of fructose undergoing first-pass intestinal metabolism in the case of the HF meal (Figure 6). As was the case for glucose appearance, the fact that the meal was not tailored to the body weight of each individual may have contributed to the variance in these data. Another limitation of our study was that insulin excursions immediately following the meal were not measured. Since insulin secretion responds to changes in portal vein glucose levels, which would be more responsive to intestinal compared to hepatic gluconeogenesis of fructose, a comparison of insulin excursions between LF and HF would have provided further insight on where the fructose was metabolized to glucose.

A single soft drink serving can contain up to 60–70 g of sugar with 36–43 g of this being fructose [27]. In comparison, our so-called HF meal contained 50 g sugar of which 27.5 g was fructose. Given the ubiquity of fructose in processed foods as well as in beverages, the amount of fructose in our meal is likely to be considerably less than that ingested during a single meal by many people in Westernized societies. In animal models, it was shown that dietary fructose is cleared by enterocytes up to a certain threshold dose that if exceeded, results in both hepatic and intestinal microbiota involvement in fructose metabolism [4]. To the extent that this spread of fructose metabolism above and beyond the enterocyte may contribute to the development of NAFLD, either directly by the unregulated hepatic catabolism of fructose [28] or indirectly through the promotion of intestinal dysbiosis [29], then it will be of utmost importance to determine the threshold intake at which this occurs in humans. Given that alterations in intestinal fructose transport and/or metabolism coupled with excessive fructose intake are associated with incidence of NAFLD as well as other complications such as insulin resistance or cardiovascular disease [30,31], it is likely that while this threshold level varies significantly between individuals, it is probably breached on a regular basis by a significant proportion of the population in Western countries.

## 5. Conclusions

In this study, we determined the contribution of dietary fructose to systemic glucose appearance and to hepatic glycogen synthesis. Had these products originated from a common pool of gluconeogenic precursors fed by fructose, then we would have expected similar gluconeogenic contributions to glucose and glycogen appearance, respectively. Instead, our data indicate that fructose was at least in part metabolized from a different precursor pool, which we postulate to be intestinal in origin. Since intestinal and hepatic pyruvate gluconeogenesis both generate the same ^2^H-enrichment pattern of plasma glucose, the precise proportion of fructose that was metabolized by the intestine versus the liver cannot be determined. Thus, in the model of fructose metabolism proposed to explain our ^2^H-enrichment data for plasma glucose and liver glycogen (Figure 6) there is the possibility for intestinal as well as hepatic gluconeogenesis. The clinical significance of our observations is that an individual’s capacity for intestinal fructose metabolism may determine their susceptibility for developing NAFLD and cardiovascular disease for a given amount of fructose intake.

## Figures and Tables

**Table 1:** Plasma metabolites measured 30 min before and 180 min after a meal containing 50 g of sugar with 55/45 fructose/glucose (High Fructose) and 5/95 fructose/glucose (Low Fructose).

	High Fructose		Low Fructose	
	−30 min	180 min	−30 min	180 min
Triglycerides (mg/dL)	77 ± 7	78 ± 6	76 ± 10	79 ± 13
NEFA (mmol/L)	0.44 ± 0.07	0.40 ± 0.09	0.35 ± 0.04	0.27 ± 0.05
Insulin (µUI/mL)	8.0 ± 1.5	5.5 ± 1.3	7.7 ± 1.2	5.7 ± 1.2 ^1^
C-peptide (ng/mL)	1.7 ± 0.2	1.9 ± 0.2	1.7 ± 0.1	2.1 ± 0.2

^1^*p* < 0.05 versus −30 min Low fructose. NEFA: non-esterified fatty acids.

**Table 2:** ^2^H-enrichment of body water, plasma glucose positions 1, 5 and 6S (G1, G5, G6*_S_*) and urinary glucuronide positions 1 and 5 (U1, U5) following ingestion of a meal containing 50 g of sugar with a 5/95 fructose/glucose proportion (Low fructose) and a meal containing 50 g of sugar with a 55/45 fructose/glucose proportion (High fructose).

	Body Water ^2^H-Enrichment	Plasma Glucose ^2^H-En-richment			Urinary Glucuronide ^2^H-Enrichment	
		G1	G5	G6*_S_*	U1	U5
High fructose	0.28 ± 0.01	0.16 ± 0.01 ^1^	0.18 ± 0.01 ^2^	0.12 ± 0.01 ^1^	0.18 ± 0.01	0.17 ± 0.01
Low fructose	0.29 ± 0.01	0.11 ± 0.01 ^1^	0.10 ± 0.01	0.07 ± 0.00	0.19 ± 0.01	0.15 ± 0.01

^1^*p* < 0.05 versus High Fructose, ^2^ *p* < 0.001 versus High Fructose.

## Data Availability

The raw data is available on request.
